# Crosstalk between the *Hp*ArsRS two-component system and *Hp*NikR is necessary for maximal activation of urease transcription

**DOI:** 10.3389/fmicb.2015.00558

**Published:** 2015-06-12

**Authors:** Beth M. Carpenter, Abby L. West, Hanan Gancz, Stephanie L. Servetas, Oscar Q. Pich, Jeremy J. Gilbreath, Daniel R. Hallinger, Mark H. Forsyth, D. Scott Merrell, Sarah L. J. Michel

**Affiliations:** ^1^Department of Microbiology and Immunology, Uniformed Services University of the Health SciencesBethesda, MD, USA; ^2^Department of Pharmaceutical Sciences, School of Pharmacy, University of MarylandBaltimore, Maryland, USA; ^3^Department of Biology, The College of William and MaryWilliamsburg, VA, USA

**Keywords:** nikR, arsRS, *pylori*, urease, *Helicobacter*, regulation, pH

## Abstract

*Helicobacter pylori* NikR (*Hp*NikR) is a nickel dependent transcription factor that directly regulates a number of genes in this important gastric pathogen. One key gene that is regulated by *Hp*NikR is *ureA*, which encodes for the urease enzyme. *In vitro* DNA binding studies of *Hp*NikR with the *ureA* promoter (*P_ureA_*) previously identified a recognition site that is required for high affinity protein/DNA binding. As a means to determine the *in vivo* significance of this recognition site and to identify the key DNA sequence determinants required for *ureA* transcription, herein, we have translated these *in vitro* results to analysis directly within *H. pylori*. Using a series of GFP reporter constructs in which the *P_ureA_* DNA target was altered, in combination with mutant *H. pylori* strains deficient in key regulatory proteins, we confirmed the importance of the previously identified *Hp*NikR recognition sequence for *Hp*NikR-dependent *ureA* transcription. Moreover, we identified a second factor, the *Hp*ArsRS two-component system that was required for maximum transcription of *ureA.* While *Hp*ArsRS is known to regulate *ureA* in response to acid shock, it was previously thought to function independently of *Hp*NikR and to have no role at neutral pH. However, our qPCR analysis of *ureA* expression in wildtype, Δ*nikR* and Δ*arsS* single mutants as well as a Δ*arsS/nikR* double mutant strain background showed reduced basal level expression of *ureA* when *arsS* was absent. Additionally, we determined that both *Hp*NikR and *Hp*ArsRS were necessary for maximal expression of *ureA* under nickel, low pH and combined nickel and low pH stresses. *In vitro* studies of *Hp*ArsR-P with the *P_ureA_* DNA target using florescence anisotropy confirmed a direct protein/DNA binding interaction. Together, these data support a model in which *Hp*ArsRS and *Hp*NikR cooperatively interact to regulate *ureA* transcription under various environmental conditions. This is the first time that direct “cross-talk” between *Hp*ArsRS and *Hp*NikR at neutral pH has been demonstrated.

## Introduction

*Helicobacter pylori* is a microaerophilic, Gram negative pathogen that infects over half of the world's human population (Marshall and Warren, 1984; Loughlin, 2003). Colonization occurs in the highly acidic gastric mucosal layer as well as at the gastric epithelial surface of the human stomach. Prolonged *H. pylori* infection is associated with the development of gastritis, peptic ulcer disease, Mucosal-Associated Lymphoid Tissue (MALT) lymphoma and gastric adenocarcinoma (Marshall and Warren, 1984; Cover and Blaser, 1992; Sepulveda and Coelho, 2002; Loughlin, 2003; Kusters et al., 2006). Current therapies for *H. pylori* infection require antibiotic cocktails of two, three or four drugs that are often not well tolerated due to adverse side effects (Loughlin, 2003; Kusters et al., 2006). If *H. pylori* infection is left untreated, colonization will persist throughout a person's lifetime (Marshall and Warren, 1984; Blaser, 1990; Cover and Blaser, 1992; Dunn et al., 1997; Sepulveda and Coelho, 2002; Loughlin, 2003). The propensity for chronic infection by *H. pylori*, coupled with the large rate of infection, manifests as a significant disease burden worldwide (Kusters et al., 2006). Thus, there is a great need to develop novel targeted anti-*Helicobacter* agents that are well tolerated (Loughlin, 2003).

While it displays optimal growth at neutral pH, *H. pylori* is one of a select number of bacteria that can survive under highly acidic conditions; this makes it ideally suited to life in the gastric mucosa. The cytosolic pH of *H. pylori* ranges from 5.3 to 7.5, and the organism can endure periodic acid shocks of pH < 2 (Wen et al., 2003, 2009; van Vliet et al., 2004a). One key feature that enables *H. pylori* to survive under such harsh conditions is its ability to convert host urea into ammonia, which serves to buffer the cytoplasmic/periplasmic pH, as well as to neutralize the immediate environment upon excretion from the bacterial cell (Wen et al., 2003; van Vliet et al., 2004a,b; Sachs et al., 2005, 2006; Scott et al., 2007). The majority of ammonia is produced by the nickel dependent urease enzyme, which converts urea to ammonia and bicarbonate. Urease accounts for approximately 10% of the total protein content of *H. pylori* (van Vliet et al., 2004a; Carter et al., 2009), and expression of the operon of genes that encode urease is known to be subject to environmental regulation.

**Figure 1:**
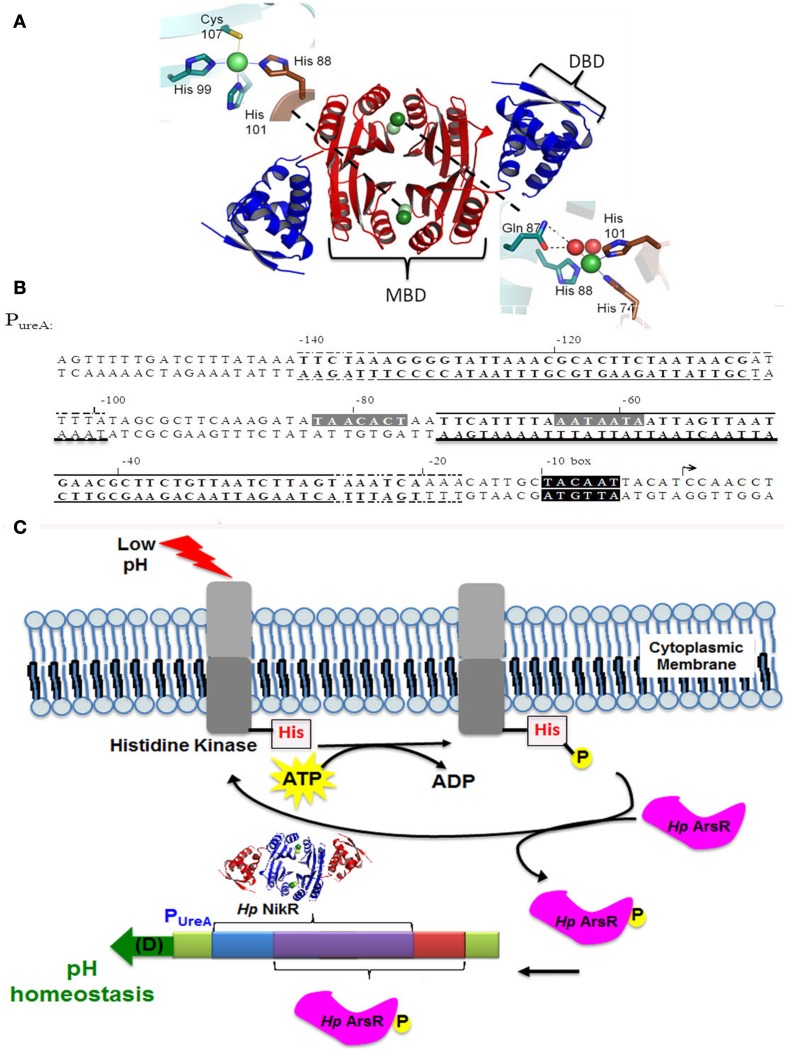
**Structure of holo-*****Hp*****NikR and architecture of the** ***ureA promoter*****. (A)** The structure of holo-*Hp*NikR. Essential areas of the protein are highlighted as follows: the metal binding domain (MBD) in red, the DNA binding domain (DBD) in blue, 4-site (top left) and 5/6-site (bottom right). This image was constructed in pymol (accession number pdb 3LGH). **(B)** The recognition sites for *Hp*NikR and *Hp*ArsR on the *H. pylori ureA* promoter. Highlighted in gray is the recognition site for *Hp*NikR, while solid and dashed lines above the sequence designate the minimum and maximum protected regions from DNase protection assays for the two distinct binding sites of *Hp*ArsR as previously described (Pflock et al., 2005). **(C)** A cartoon demonstrating the operator overlap at the *ureA* promoter for *Hp*NikR (pdb 3LGH) and *Hp*ArsR within the context of the biological role of *Hp*ArsR. The region colored purple represents the overlapping promoter sites.

The nickel dependent metalloregulatory protein, *Hp*NikR, is known to be a key regulator of urease expression (van Vliet et al., 2002; Contreras et al., 2003; Ernst et al., 2005; Dosanjh and Michel, 2006; Maier et al., 2007). *Hp*NikR functions as a tetrameric metalloregulatory protein. Each *Hp*NikR monomer is comprised of two domains, named the N-terminal and C-terminal domains (West et al., 2010, 2012; Benini et al., 2011). To form the functional tetrameric protein, all four C-terminal domains form a central tetrameric metal binding domain (MBD), which serves as the site of nickel coordination. The MBD is then flanked on either side by two N-terminal domains that fold to form two dimeric DNA binding domains (DBD). The DBDs adopt a classic ribbon-helix-helix fold, which is commonly found in DNA binding proteins (Figure 1A) (Chivers and Sauer, 1999, 2000, 2002; Chivers and Tahirov, 2005; Schreiter and Drennan, 2007; West et al., 2010).

*Hp*NikR requires Ni(II) coordination to the MBD in order to recognize and bind to DNA via the DBD (Abraham et al., 2006; Benanti and Chivers, 2007; Dosanjh et al., 2007; Bahlawane et al., 2010). Crystallographic studies conducted by one of our laboratories have shown that Ni(II) coordinates to two distinct sites at the MBD of *Hp*NikR: two Ni(II) ions coordinate at square planar sites that utilize a 3 His/1Cys ligand set and two Ni(II) ions coordinate to a square pyramidal/octahedral sites that utilize a 3 His/ 2–3 H_2_O ligand set (Figure 1A) (West et al., 2010, 2012). This heterogeneous nickel coordination controls the overall flexibility of the DBDs to favor high affinity DNA binding (West et al., 2012).

*Hp*NikR is a global regulator of transcription in *H. pylori* (Contreras et al., 2003; van Vliet et al., 2004a; Ernst et al., 2005; Abraham et al., 2006). In addition to urease, *Hp*NikR activates and represses the expression of at least forty genes in response to nickel availability (van Vliet et al., 2002; Contreras et al., 2003; Ernst et al., 2005, 2006; Abraham et al., 2006; Davis et al., 2006; Dosanjh and Michel, 2006; Benanti and Chivers, 2007, 2011; Dosanjh et al., 2007, 2009; Zambelli et al., 2008; Li and Zamble, 2009; Muller et al., 2011; Romagnoli et al., 2011; Evans and Michel, 2012; Jones et al., 2015). Genes which have been shown to be directly regulated by *Hp*NikR encode for proteins that regulate nickel uptake (*nixA, frpB4, fecA3, frpB2*, *ceuE*), storage (*hpn, hpn-like*) and regulation (*nikR*), genes involved in iron uptake (*exbB*) and regulation (*fur*), genes involved in infection (*hspA*), and genes that encode for outer membrane proteins (*exbB/tonB*) (van Vliet et al., 2002; Contreras et al., 2003; Delany et al., 2005; Ernst et al., 2005, 2006; Abraham et al., 2006; Davis et al., 2006; Benanti and Chivers, 2007; Dosanjh et al., 2007, 2009; Jones et al., 2015). The direct binding of *Hp*NikR to the promoter sequences of these genes has been shown via DNase footprinting, electrophoretic mobility shift assays (EMSA), fluorescence anisotropy (FA) and/or isothermal titration calorimetry (ITC) (Contreras et al., 2003; Delany et al., 2005; Ernst et al., 2005, 2006; Abraham et al., 2006; Davis et al., 2006; Benanti and Chivers, 2007, 2011; Dosanjh et al., 2007, 2009; Zambelli et al., 2008; Li and Zamble, 2009; Muller et al., 2011; Romagnoli et al., 2011; Jones et al., 2015). In addition, Krezel and co-workers recently reported a direct role for the RNA polymerase alpha-subunit C-terminal domain in promoting *Hp*NikR/*ureA* binding (Borin et al., 2014). From those studies, a general model for *Hp*NikR gene regulation has been formulated. In this model, *Hp*NikR activates transcription by binding upstream of the RNA polymerase binding site, which aids in the recruitment of RNA polymerase (i.e., for *ureA*). In contrast, *Hp*NikR represses transcription by a simple steric hindrance mechanism in which *Hp*NikR blocks the interaction of RNA polymerase with the promoter by binding at promoter sequences overlapping the −10 or −35 hexameric boxes (e.g., *nikR, nixA, fur, frpB4, exbB, fecA3*) (van Vliet et al., 2002; Delany et al., 2005; Ernst et al., 2006; Wolfram et al., 2006; Danielli et al., 2009; Dosanjh et al., 2009). In addition to the genes for which direct protein/DNA binding has been established, approximately 30 additional genes have been predicted to be regulated by *Hp*NikR (Contreras et al., 2003; Delany et al., 2005; Ernst et al., 2005; Abraham et al., 2006; Davis et al., 2006; Ernst et al., 2006; Benanti and Chivers, 2007; Dosanjh et al., 2007; Zambelli et al., 2008; Dosanjh et al., 2009; Li and Zamble, 2009; Benanti and Chivers, 2011; Muller et al., 2011; Romagnoli et al., 2011). Whether these additional genes are regulated directly or indirectly has yet to be established. These genes include components involved in motility (*cheV, flaA, flaB*) and stress response (*hrcA, grpE, dnaK*), as well as outer membrane proteins (*omp11, omp31, omp32*) (Contreras et al., 2003; Dosanjh et al., 2009).

The promoters for the genes that are directly regulated by *Hp*NikR share common sequences, but are not identical. These DNA targets share a partially palindromic sequence composed of two sets of seven base pairs (half-sites), separated by eleven bases (Delany et al., 2005; Dosanjh et al., 2007, 2009; Stoof et al., 2010; Evans and Michel, 2012). *Hp*NikR binds to a subset of the promoters with high affinity (*K_d_* ~ nM) and a subset of the promoters with low affinity (*K_d_* ~ μM) (Dosanjh et al., 2009). The promoter sequences for which high affinity recognition is measured are from genes that encode for nickel regulated proteins (*ureA, nixA, frpb4, fecA3*), while the promoter sequences for which low affinity binding has been measured are from genes that encode for other proteins (*fur, nikR, exbB*) (Dosanjh et al., 2009). Thus, *in vitro Hp*NikR preferentially recognizes genes that encode for nickel-regulated proteins. The DNA target sequences for which high affinity DNA binding is observed have greater conservation of sequence at the second half-site (Evans and Michel, 2012). Studies using the *ureA* promoter in which the DNA sequences have been systematically altered have identified key bases within the second half-site that are essential for high-affinity protein/DNA binding (Delany et al., 2005; Dosanjh et al., 2009; Evans and Michel, 2012). In addition, the intact partial-palindrome is required for high affinity DNA binding by *Hp*NikR *in vitro* (Dosanjh et al., 2009). When either half of the partial palindrome was modified to all cytosines, the affinity of *Hp*NikR for the *ureA* promoter decreased from 8.0 ± 1 nM to 1000 ± 94 nM for WT/C (CTTCAAAGATA**TAACACT**AATTCATTTTA**CCCCCCC**ATTAGTTAATGA) and 4900 ± 780 nM for C/WT (CTTCAAAGATA**CCCCCCC**AATTCATTTTA**AATAATA**ATTAGTTAATGA) (Dosanjh et al., 2009; West et al., 2012). When both halves of the palindrome were modified, DNA binding was fully abrogated (Dosanjh et al., 2009).

The *Hp*ArsRS two-component system has also been shown to regulate a wide variety of genes in *H. pylori.* These genes include those that encode for proteins involved in acid resistance, (including urease), acetone metabolism (acetone carboxylase), resistance to oxidative stress (thioredoxin reductase), and quorum sensing (Pflock et al., 2005, 2006a,b; Wen et al., 2007; Goodwin et al., 2008; Loh et al., 2010). Within the regulatory pathway, *Hp*ArsS serves as a sensor protein that phosphorylates the *Hp*ArsR response regulator. Phosphorylation occurs via a two-step process: *Hp*ArsS autophosphorylates at histidine 214 and then transfers the phosphate to aspartic acid 52 on *Hp*ArsR to generate *Hp*ArsR-P (Schar et al., 2005; Pflock et al., 2006a; Joseph and Beier, 2007; Gupta et al., 2009; Muller et al., 2009). HpArsR appears essential for bacterial viability and binds to different promoter elements based on the phosporylation state. Binding at *P_ureA_* requires phosphorylation and the binding site of *Hp*ArsR-P overlaps with the binding site recognized by *Hp*NikR (Pflock et al., 2005) (Figures 1B,C). Given that *Hp*ArsR and *Hp*NikR respond to different environmental conditions, acid and nickel, respectively, these two regulators are believed to function independently of one another (Pflock et al., 2005).

Most of the studies that have investigated binding of *Hp*NikR to target promoters have been conducted *in vitro*. However, it is clear that the cytoplasm of the bacterial cell is a much more complex environment than the one modeled in an *in vitro* experiment. There is one *in vivo* study of *Hp*NikR mediated nickel response in which quantitative real-time PCR was used to measure *Hp*NikR regulation of target genes (Muller et al., 2011). In this study, a complex relationship between Ni(II) availability and activation or repression of a series of genes was determined, suggesting that *Hp*NikR activity *in vivo* may be more complex than observed *in vitro*. The goal of the studies described herein was to determine if the DNA sequences required for recognition of *P_ureA_* by *Hp*NikR are the same *in vitro* and *in vivo*. To this end, we constructed transcriptional reporters in which the wildtype *ureA* promoter or mutant versions of the promoter were fused to the gene encoding GFP. Reporter assays were then used to monitor *ureA* promoter activity directly in *H. pylori*. The wildtype *P_ureA_* sequence exhibited high levels of GFP expression that increased with increasing concentrations of nickel. Unexpectedly, mutation of the half-sites did not prevent basal level urease expression, but negated the Ni(II) dependence of *ureA* expression. These results suggested that another factor rescues *P_ureA_* transcription when the DNA target sequence for *Hp*NikR is compromised. Herein, we show that the *Hp*ArsRS acid response regulatory system, which has previously been shown to regulate *ureA* expression in response to low pH, also affects *ureA* transcription at neutral pH. This is the first time that *Hp*ArsR has been shown to regulate urease expression in conjunction with *Hp*NikR, and we propose that there is a cooperative interaction between these two regulators to control urease expression in *H pylori*.

## Materials and methods

### Bacterial strains and growth

Primer sequences are listed in Table 1 and bacterial strains and plasmids used in this study are listed in Table 2. The *H. pylori* strains used in this study are all derivatives of G27 (Covacci et al., 1993; Baltrus et al., 2009). *H. pylori* strains were maintained as frozen stocks at −80°C in brain heart infusion broth (BD Biosciences) supplemented with 10% fetal bovine serum (Gibco) and 20% glycerol (EMD chemicals, Inc.). Bacterial strains were cultured essentially as previously described (Carpenter et al., 2007). Briefly, strains were grown on horse blood agar (HBA) plates that contained 4% Columbia agar base (Neogen Corporation), 5% defibrinated horse blood (HemoStat Laboratories, Dixon, CA), 0.2% β-cyclodextrin (Sigma), 10 μg/ml vancomycin (Amresco), 5 μg/ml cefsulodin (Sigma), 2.5 U/ml polymyxin B (Sigma), 5 μg/ml trimethoprim (Sigma), and 8 μg/ml amphotericin B (Amresco). Liquid cultures of *H. pylori* were grown in brucella broth (Neogen Corporation) supplemented with 10% fetal bovine serum and 10 μg/ml vancomycin at 37°C with shaking at 100 rpm. As noted in Table 2, where appropriate, cultures and plates were supplemented with 8 μg/ml chloramphenicol (Cm) (EMD Chemicals, Inc.) and/or 25 μg/ml kanamycin (Kan) (Gibco). In addition, where detailed below, some HBA plates contained 5% sucrose (Suc) (Sigma). Both liquid and plate cultures were grown under microaerobic conditions (5% O_2_, 10% CO_2_, and 85% N_2_) generated with an Anoxomat gas evacuation and replacement system (Spiral Biotech) in gas evacuation jars. Exponential phase cultures were grown in liquid culture for 20 h, while stationary phase cultures were grown for 44 h.

**Table 1:** **List of oligonucleotides used in this study**.

**Name**	**Sequence**	**(5′–3′) site**	**References**
**SOE PRIMERS *ureA* PROMOTER MUTANTS**			
UreA_F_Prom_KpnI	GGTACCCAAAAACAAAACAAAATTAAGGCATA	KpnI	This study
UreA_R_Prom_XbaI	TCTAGATGGGGTGAGTTTCATCTCATT	XbaI	This study
F1_ureA_prom_switch	AAATACCCCCCCAATTCATTTTAAATAATAATTAGTTAATGAACGCTTCTGTTAATCTT		This study
R1_ureA_prom_switch	TAATTATTATTTAAAATGAATTGGGGGGGTATTTTTGAAGCGCTATAAAAGCGTTA		This study
F2_ureA_prom_switch	AAATATAACACTAATTCATTTTACCCCCCCATTAGTTAATGAACGCTTCTGTTAATCTT		This study
R2_ureA_prom_switch	TAATGGGGGGGTAAAATGAATTTATTATTTATTTTTGAAGCGCTATAAAAGCGTTA		This study
F3_ureA_prom_switch	AAATACCCCCCCAATTCATTTTACCCCCCCATTAGTTAATGAACGCTTCTGTTAATCTT		This study
R3_ureA_prom_switch	TAATGGGGGGGTAAAATGAATTGGGGGGGTATTTTTGAAGCGCTATAAAAGCGTTA		This study
**SOE PRIMERS NikR DELETION**			
U1338-F	CCAAGCACTGCAAAAACAAA		This study
U1338-R	TTCTAGTTGCAAGCGTTGGACCCGGGAGGCTCGAGTGGGTGTATCCATTGAGAAAAA	SmaI, XhoI	This study
D1338-F	TTTTTCTCAATGGATACACCCACTCGAGCCTCCCGGGTCCAACGCTTGCAACTAGAA	SmaI, XhoI	This study
D1338-R	GCCCTTTCTTGCTTGATTTC		This study
**SOE PRIMERS ArsS DELETION**			
HP0165_Up_F	AAGTGTGTAGGCGCATTTCC		This study
HP0165_Up_R	ATCTTCTCAATCGTTTGAACATGTTCTCTCTAACCCCTTAACTCCTTATTAGAATCA		This study
HP0165_Down_F	TGATTCTAATAAGGAGTTAAGGGGTTAGAGAGAACATGTTCAAACGATTGAGAAGAT		This study
HP0165_Down_R	CGCTTTCAGCCAAAATAAGC		This study
**SOE PRIMERS *ureA* PROMOTER DELETION**			
ureA_Up_F_Prom_KanSacB	GCGTTTTCCTTGCTCAGTTT		This study
ureA_Up_R_Prom_KanSacB	CTCTTTTGGGGTGAGTTTCATCCCGGGAGGCTCGAGTTATGCCTTAATTTTGTTTTG	SmaI, XhoI	This study
ureA_Down_F_Prom_KanSacB	CAAAACAAAATTAAGGCATAACTCGAGCCTCCCGGGATGAAACTCACCCCAAAAGAG	SmaI, XhoI	This study
ureA_Down_R_Prom_KanSacB	AGTCCCATCAGGAAACATCG		This study
ureA_Up_R_Prom_complementation	CCTTTATTTTAAAAAGAGTGATTATGCCTAATTTTGTTTTGTTTTTG		This study
ureA_Down_F_Prom_complementation	GGAAAAACACTTTAAGAATAGGAGAATGAGATGAAACTCACCCCA		This study
**qRT-PCR PRIMERS**			
ureA qPCR F	GAAGAAGCGAGAGCTGGTAAA		This study
ureA qPCR R	AGATGATGTGATGGATGGCG		This study
G27_16SRT-F	ATGGATGCTAGTTGTTGGAGGGCT		^a^
G27_16S RT-R	TTAAACCACATGCTCCACCGCTTG		^a^
***Hp* ArsR-HIS PROTEIN EXPRESSION AND PURIFICATION**			
arsR Fwd.Bam	CCCGGATCCATGATAGAAGTTTTAATGATAGAAG BamHI		This study
arsR Rev.HindIII	CCCAAGCTTTCAGTATTCTAATTTATAACCAATCCCTC HindIII		This study
***Hp* ArsR-HIS FLOURESENCE ANISOTROPY TARGETS**			
PureA-F	CTTCAAAGATA**TAACACT**AATTCATTTTA**AATAATA**ATTAGTTAATGA		b, c, d, e
PureA Wt/C	CTTCAAAGATA**TAACACT**AATTCATTTTA**CCCCCCC**ATTAGTTAATGA		c
PureA C/Wt	CTTCAAAGATA**CCCCCCC**AATTCATTTTA**AATAATA**ATTAGTTAATGA		c
PureA C/C	CTTCAAAGATA**CCCCCCC**AATTCATTTTA**CCCCCCC**ATTAGTTAATGA		c

a Gilbreath et al. (2012).

b Dosanjh and Michel (2006).

c Dosanjh et al. (2009).

d Evans and Michel (2012).

e West et al. (2012).

Bold print indicates the sequence within the WT ureA promoter that was chosen for mutation.

The underlined sequences correspond to the restriction sites for the enzymes listed in the same row.

**Table 2:** **List of strains used in this study**.

**Plasmid or Strain**	**Description**	**References**
**PLASMIDS**		
pDSM278	pGEM T-easy::*cat*	This study
pDSM462	pGEM T-easy::WT *ureA* promoter	This study
pDSM923	pGEM T-easy::upstream region of *nikR* fused to downstream region	This study
pDSM924	pGEM T-easy::Δ*nikR*::*cat*	This study
pDSM922	pBluescript::Δ*arsS::kan-sacB*	This study
pDSM1070	pGEM T-easy::upstream region of *arsS* fused to downstream region	This Study
pDSM199	pTM117::promoterless	Carpenter et al., 2007
pDSM463	pTM117::WT *ureA* promoter	This study
pDSM697	pTM117:: *ureA* promoter mutant C/C	This study
pDSM698	pTM117:: *ureA* promoter mutant C/WT	This study
pDSM796	pTM117:: *ureA* promoter mutant WT/C This study	
**STRAINS**		
***H. pylori* strains**		
G27	WT	Baltrus et al., 2009
DSM215	G27 (pTM117::promoterless), Kan^r^	This study
DSM464	G27 (pTM117::WT *ureA* promoter), Kan^r^	This study
DSM763	G27 (pTM117:: *ureA* promoter mutant C/C), Kan^r^	This study
DSM764	G27 (pTM117:: *ureA* promoter mutant C/WT), Kan^r^	This study
DSM797	G27 (pTM117:: *ureA* promoter mutant WT/C), Kan^r^	This study
DSM975	G27 Δ*nikR::cat*, Cm r	This study
DSM980	G27 Δ*nikR::cat* (pTM117::promoterless), Cm^r^, Kan^r^	This study
DSM976	G27 Δ*nikR::cat* (pTM117::Wt *ureA* promoter), Cm^r^, Kan^r^	This study
DSM977	G27 Δ*nikR::cat* (pTM117:: *ureA* promoter mutant C/C), Cm^r^, Kan^r^	This study
DSM978	G27 Δ*nikR::cat* (pTM117:: *ureA* promoter mutant C/WT), Cm^r^, Kan^r^	This study
DSM979	G27 Δ*nikR::cat* (pTM117:: *ureA* promoter mutant WT/C), Cm^r^, Kan^r^	This study
DSM983	G27 Δ*arsS* markerless	This study
DSM1069	G27 Δ*arsS::kan-sacB*, Kan^r^, Suc^s^	This study
DSM1071	G27 Δ*arsS/nikR::cat*, Cm^r^	This study
DSM1398	G27 Δ*arsS* markerless (pTM117::promoterless), Kan^r^	This study
DSM1399	G27 Δ*arsS* markerless (pTM117::WT *ureA* promoter), Kan^r^	This study
DSM1400	G27 Δ*arsS* markerless (pTM117:: *ureA* promoter mutant C/C), Kan^r^	This study
DSM1401	G27 Δ*arsS* markerless (pTM117:: *ureA* promoter mutant C/WT), Kan^r^	This study
DSM1402	G27 Δ*arsS* markerless (pTM117:: *ureA* promoter mutant WT/C), Kan^r^	This study
DSM1403	G27 Δ*arsS/nikR*::*cat* (pTM117::promoterless), Cm^r^, Kan^r^	This study
DSM1404	G27 Δ*arsS/nikR*::*cat* (pTM117::WT *ureA* promoter), Cm^r^, Kan^r^	This study
DSM1405	G27 Δ*arsS/nikR*::*cat* (pTM117:: *ureA* promoter mutant C/C), Cm^r^, Kan^r^	This study
DSM1406	G27 Δ*arsS/nikR*::*cat* (pTM117:: *ureA* promoter mutant C/WT), Cm^r^, Kan^r^	This study
DSM1407	G27 Δ*arsS/nikR*::*cat* (pTM117:: *ureA* promoter mutant WT/C), Cm^r^, Kan^r^	This study

### Construction of a Δ*nikR::cat H. pylori* strain

The *HpnikR* mutant strain was constructed using a strategy that resulted in replacement of the *HpnikR* sequence with the *cat* gene, which encodes for chloramphenicol resistance. Briefly, a 968 bp fragment containing the region directly upstream of *nikR* was amplified with primers U1338-F and U1338-R, the latter of which was engineered to contain SmaI and XhoI restriction sites. This fragment was then fused via splicing by overlap extension (SOE) PCR to a 1098 bp fragment containing the region immediately downstream of *nikR*, which was amplified with primers D1338-F and D1338-R, the former of which contains SmaI and XhoI restriction sites. The SOE product was cloned into pGEM-T Easy to create pDSM923. Sequence analysis showed that only the SmaI site was preserved in the SOE fusion product. The *cat* gene (Carpenter et al., 2007), which had first been cloned into pGEM-T Easy as pDSM278, was liberated via restriction digestion with EcoR1 New England Biolabs (NEB), Klenow (NEB) treated, and then ligated with the SmaI (NEB) digested and calf intestine phosphatase (NEB) treated, pDSM923; the resulting *nikR* deletion construct was named pDSM924. pDSM924 was subsequently transformed into G27, and transformants were selected for on HBA plates containing 8 μg/mL Cm. Resulting colonies were screened for differences in size in the *nikR* region via PCR with the U1338-F and D1338-R primers. For those colonies showing the expected change in size, the PCR product was next sequenced to verify deletion of *nikR*. One such colony showing a deletion insertion of *nikR* was named DSM975.

### Construction of a markerless Δ*arsS H. pylori* strain

The *arsS* mutant strain was created using pDSM922, which is a suicide vector that contains a counter selectable marker (generous gift of Liz Marcus and David Scott, UCLA School of Medicine). Briefly, the plasmid contains a *kan-sacB* counter selectable cassette, previously described by Copass et al. (1997), that is flanked by the 600 and 400 bp immediately upstream and downstream, respectively, of HP0165 (*arsS*). These regions were originally amplified using strain 43,504 as the template (Marshall and Goodwin, 1987). pDSM922 was naturally transformed into G27, and transformants were selected on HBA plates containing 25 μg/ml kanamycin. The resulting transformants were patched on HBA plates containing 5% sucrose to ensure sucrose sensitivity, and deletion insertion of *arsS* was then confirmed by PCR amplification of the region with HP0165_up_F and HP0165_down_R primers followed by sequencing with the same primers. One such Δ*arsS* mutant was named DSM1069, which then served as the background strain to create the markerless mutant.

To create the markerless deletion strain, the region immediately upstream and downstream of *arsS* were fused together via SOE PCR; the upstream region was amplified with the HP0165_Up_F and HP0165_Up_R primers, the downstream region was amplified with the HP0165_Down_F and HP0165_Down_R primers, and the products from these reactions were gel purified and fused together via SOE PCR using primers (HP0165_Up_F and HP0165_Down_R). The resulting product was gel purified, and naturally transformed into DSM1069. Transformants were selected on HBA plates containing 5% sucrose and then patched onto HBA plates containing 25 μg/ml kanamycin to ensure kanamycin sensitivity; double crossover homologous recombination resulted in the replacement of the *kan-sacB* cassette to create a markerless deletion of the *arsS* gene. Proper integration was confirmed by PCR and sequencing with the HP0165_Up_F and HP0165_Down_R primers. The resulting strain was named DSM983.

### Construction of a Δ*arsS/nikR::cat H. pylori* strain

The Δ*arsS/nikR::cat* mutant strain was created by naturally transforming pDSM924, the *nikR::cat* deletion construct, into *H. pylori* strain DSM983. Transformants were then screened on HBA plates supplemented with 8 μg/mL Cm to ensure chloramphenicol resistance. Proper integration was confirmed first by PCR with the U_1338_F and D_1338_R primers. For those colonies showing the expected change in size for the *nikR* gene, the PCR product was sequenced to verify deletion of *nikR*. To ensure that the Δ*arsS* markerless deletion was still intact for this strain, PCR with the HP0165_Up_F and HP0165_Down_R primers followed by sequencing of the PCR product was performed. The resulting strain was named DSM1071.

### Construction of *ureA*::GFP transcriptional fusions

Transcriptional fusions to the promoterless *gfpmut3* allele carried on pTM117 (Carpenter et al., 2007) were constructed to monitor *ureA* expression. Briefly, the wildtype *ureA* promoter was PCR amplified from G27 using the UreA_F_Prom_KpnI and UreA_R_Prom_XbaI primers; these primers incorporate KpnI and XbaI restriction sites, respectively. The resulting PCR fragment was subcloned into pGEM-T Easy (Promega) to create pDSM462. The *ureA* promoter was then removed via digestion with KpnI (NEB) and XbaI (NEB) and ligated into the appropriately digested pTM117 vector to create pDSM463. The proper fusion was confirmed by PCR amplification with the UreA_F_Prom_KpnI and UreA_R_Prom_XbaI primers and by sequencing with the same primers. pDSM463 was then naturally transformed into G27, DSM975, DSM983 and DSM1071, and transformants were selected on HBA plates containing 25 μg/ml Kan. The individual strains transformed with pDSM463 are described in Table 2.

Mutant *ureA* promoter constructs were each created using SOE and primer pairs that incorporated the desired mutation during the process of amplification. Specifically, the C/WT mutant promoter was created using the primer pairs F1_ureA_prom_switch and UreA_R_Prom_XbaI, and UreA_F_Prom_KpnI and R1_ureA_prom_switch. The WT/C mutant promoter was created using the primer pairs F2_ureA_prom_switch and UreA_R_Prom_XbaI, and UreA_F_Prom_KpnI and R2_ureA_prom_switch. Finally, the C/C *ureA* promoter mutant was created using the primer pairs F3_ureA_prom_switch and UreA_R_Prom_XbaI, and UreA_F_Prom_KpnI and R3_ureA_prom_switch. Each of the mutant *ureA* promoters were subcloned into pGEM-T Easy (Promega), removed by digestion with KpnI (NEB) and XbaI (NEB), and ligated into the appropriately digested pTM117 vector. The constructed plasmids are pDSM697 (C/C), pDSM698 (C/WT), and pDSM796 (WT/C). All fusions were confirmed by PCR amplification with UreA_F_Prom_KpnI and UreA_R_Prom_XbaI primers and by sequencing with the same primers. Each of the resulting plasmids containing the individual *ureA* promoter mutations as well as a promoterless GFP fusion vector (pDSM199) were naturally transformed into G27, DSM975, DSM983, and DSM1071, and transformants were selected on HBA plates containing 8 μg/ml Cm and 25 μg/ml Kan (DSM 975 and DSM 1071), or 25 μg/ml Kan alone (G27 and DSM 983).

### GFP reporter assays

The ability of the *ureA* transcriptional fusions to drive expression of GFP was assessed visually utilizing an Olympus BX61 fluorescent microscope, as well as using flow cytometry as previously described (Carpenter et al., 2007). Briefly, strains containing the *ureA* promoter fusions were grown overnight in liquid cultures containing varying NiSO_4_ concentrations (0, 0.5, 1.0, 10 μM) (Sigma). Following overnight growth, 0.5–1.5 ml of each culture was pelleted and resuspended in 1–2 ml of sterile 1× phosphate-buffered saline depending on the density of the culture. Bacterial clumps and culture debris were subsequently removed by passing the resuspended culture through a 1.2-μm Acrodisc PSF syringe filter (Pall). Flow cytometry analysis for the *ureA* fusion constructs was performed using either a Beckman Coulter Epics XL-MCL flow cytometer with a laser setting of 750 V or a BD SLR II flow cytometer. 20,000 events were collected for each assay. WinList 3D, version 6.0 (Verity Software House) and FlowJo, version X (FLOWJO, LLC) were used to analyze the flow cytometry data. These experiments were performed 3–5 times for each strain-reporter plasmid combination.

### RNA isolation, cDNA synthesis and RT-PCR

In addition to the GFP reporter assays, RT-PCR was utilized to measure *ureA* expression under normal, 10 μM nickel supplemented, low pH (pH 5.0), and combined nickel supplementation and low pH conditions. Bacterial liquid cultures of DSM1 (WT G27), DSM975 (Δ*nikR*), DSM983 (Δ*arsS*), and DSM1071 (Δ*arsS/nikR*) were grown for 18 h, and then each culture was divided into four equal portions. The first portion was utilized for RNA isolation and represents the normal media sample. The remaining portions were pelleted and resuspended in one of the following supplemented liquid culture medias: 10 μM NiSO_4_(Sigma-Aldrich), pH 5.0 (achieved through the addition of HCl to the media), or pH 5.0 with 10 μM NiSO_4_. These portions of each culture were then maintained for another 90 min prior to RNA isolation. RNA isolation was performed as previously described (Thompson et al., 2003). The integrity of the RNA was determined through visualization on agarose gels. Next, cDNA was generated as previously described (Gilbreath et al., 2012) using the Quantitect reverse transcriptase kit (Qiagen) according to the manufacturer's protocol. Control reactions for each sample were also performed without the addition of reverse transcriptase (noRT) enzyme. Following cDNA synthesis, quantitative real-time PCR (qPCR) for *ureA* as well as the 16S internal reference gene was performed using the primers listed in Table 1. qPCR was conducted similar to the methods used by Gilbreath et al. (2012). Briefly, qPCR reaction mixtures composed of 1x Roto-Gene SYBR green RT-PCR master mix, 3 pmol each of forward and reverse primer pair, and 1 μL of either cDNA or noRT reaction for use as template were combined and brought to a total volume of 20 μL with water. The following 2-step cycling conditions were used: 5 min at 95°C (initial activation) followed by 35 cycles of 5 s at 95°C (denaturation) and 10 s at 50°C (annealing/extension); SYBR green fluorescence was measured at each extension step. Relative gene expression was calculated using the 2^−ΔΔCT^ method. Post-run melt curve analysis were performed to ensure specificity of amplification. Four biologically independent replicates of these experiments were conducted.

### *H. pylori Hp*ArsR cloning, expression and purification

PCR primers were designed to amplify the *arsR* gene from *H. pylori* J99 with BamH1 and HindIII restriction sites included. The *arsR* gene was ligated into a pQE3 vector (Qiagen), which includes an N-terminus hexa-histidine coding sequence. The identity of the cloned *arsR* gene was confirmed by DNA sequencing. For protein expression, *arsR*-pQE3 was transformed into *E. coli* M15-pREP4 cells, and grown in LB medium containing ampicillin (100 μg/mL) and kanamycin (20 μg/mL). When the culture reached an *A*_600_ of 0.6, protein expression was induced for 4 h with 1 mM isopropyl-β-d-thiogalactopyranoside (IPTG). Cells were collected by centrifugation at 12,000 × g for 10 min. All buffers utilized in the *Hp*ArsR studies contained 5 mM tris(2-carboxyethyl) phosphine hydrochloride (TCEP) to prevent oligomerization of the protein. Cell pellets were resuspended in 20 mM Tris-HCl, 500 mM NaCl, 5 mM TCEP, 1 mM phenylmethylsulfonyl fluoride (PMSF) pH 7.5 and then lysed by sonication on ice. After sonication, the preparation was centrifuged at 31,000 × g for 20 min. The supernatant (~ 100 ml) was collected and applied to a 30-ml metal affinity chromatography column (His-Bind, Novagen) charged with Ni(II). The column was washed with a 50 mM imidazole gradient to remove proteins bound non-specifically to the column, and the protein of interest, including the hexa-histidine tag, was eluted with 250 mM imidazole over 45 ml. Five milliliter fractions corresponding to the elution were collected, and the purity of the proteins was visually assessed using 15% SDS-PAGE gels stained with coomasie. Fractions determined to be >95% pure were pooled, and buffer exchanged into 50 mM Tris-HCl, 5 mM MgCl_2_, 50 mM KCl, 5 mM TCEP, pH 7.5 and concentrated to a volume of 6 ml using Amicon Ultra-15 centrifugal filters [5-kDa molecular weight cut-off (MWCO) membrane] for use in DNA binding assays.

### *In vitro* phosphorylation of *Hp*ArsR

*Hp*ArsR was phosphorylated (*Hp*ArsR-P) *in vitro* by incubating the protein in phosphorylation buffer (50 mM Tris-HCl, 5 mM MgCl_2_, 50 mM KCl, 5 mM TCEP, pH 7.5) with 50 mM acetylphosphate for 60 min at 25°C (McCleary and Stock, 1994; Dietz et al., 2002). Phosphorylation yields were not independently measured. Post-phosphorylation, the KCl concentration was adjusted to 100 mM to be consistent with past salt concentrations used in the studies of *Hp*NikR DNA recognition (Dosanjh et al., 2007, 2009; West et al., 2012).

### Oligonucleotide probes

HPLC-purified oligonucleotide probes were purchased from Integrated DNA Technologies (Coralville, IA) and were either labeled with fluorescein (F) or unlabeled as indicated in Table 1. Upon receipt, the probes were resuspended in DNase-free water and quantified. To obtain double stranded probes, each oligonucleotide probe was mixed with a probe with the complementary sequence such that there was a 1.25:1 ratio of unlabeled to labeled oligonucleotide probe in annealing buffer (10 mM Tris, 10 mM NaCl at pH 8.0). The annealing reaction mixtures were placed in a water bath set to a temperature 10°C higher than the melting temperatures (*T*ms) of the component oligonucleotides. The water bath was then immediately turned off, and the annealing reaction mixtures were allowed to cool overnight. Double-stranded oligonucleotides were quantified and stored at −20°C (Dosanjh et al., 2007, 2009; West et al., 2012).

### Fluorescence anisotropy monitored titrations of *Hp*ArsR-P and *Hp*ArsR with P_*ureA*_

A fluorescence anisotropy (FA) assay was used to characterize the interaction of *Hp*ArsR-P and *Hp*ArsR with the *ureA* promoter and related mutants. Measurements were taken on an ISS PC-1 spectrofluorimeter configured in the L format with an excitation wavelength of 495 nm and an emission wavelength of 519 nm. The band-pass for excitation was 2 nm and 1 nm for emission. 15 nM of *P_ureA_* in 50 mM Tris-HCl, 5 mM MgCl_2_, 100 mM KCl, 5 mM TCEP, pH 7.5 was added to a cuvette that had been pretreated with 5 mM bovine serum albumin (BSA) to prevent adherence of either the protein or DNA to the cuvette walls.

*Direct titrations:* For direct titrations, either *Hp*ArsR-P or *Hp*ArsR was titrated into fluorescein labeled ureA (*ureA-F)* and the change in anisotropy as a function of added protein was measured. The data were analyzed by converting the anisotropy to fraction bound, F_bound_ (the fraction of *Hp*ArsR-P bound to DNA at a given DNA concentration), using the equation (Lakowicz, 1999):

$$F_{bound}=\frac{r-r_{free}}{\left(r_{bound}-r)Q+(r-r_{free}\right)}$$

Where *r_free_* is the anisotropy of the fluorescein-labeled oligonucleotide, *r_bound_* is the anisotropy of the DNA/protein complex at saturation, and Q, is the quantum yield ratio of the bound to the free form and is calculated from the fluorescence intensity changes that occur (*Q = I_bound_/I_free_*). The typical Q for *Hp*ArsR-P DNA binding experiments was *Q* = 0.87. *F_bound_* was plotted against the protein concentration and fit using a one-site binding model:

$$\begin{array}{l} \;P+D⇌PD\\ \;K_{d}=\frac{\left[P][D\right]}{\left[PD\right]}\\ F_{bound}=\frac{\begin{array}{l} P_{total}+D_{total}+K_{d}-\sqrt{{\left(P_{total}+D_{total}+K_{d}\right)}^{2}-4P_{total}D_{total}} \end{array}}{2D_{total}} \end{array}$$

Where P is the protein concentration and D is the DNA concentration. Each data point from the FA assay represents the average of 31 readings taken over a time course of 100 s. Each titration was carried out in triplicate.

#### Competitive titrations

For competitive titrations, an unlabeled DNA oligomer was titrated into a solution containing 1500 nM *Hp*ArsR-P and 5 nM *P_ureA_-F* and the decrease in anisotropy (r) as the unlabeled DNA oligomer competed with the labeled oligonucleotide was recorded. The resultant anisotropy values were converted to fraction bound. The competition experiments were performed with *Hp*ArsR-P concentrations at levels near saturation to minimize the amount of unlabeled DNA required to complete the titrations. Experiments were performed aerobically as no difference in binding was observed between experiments performed anaerobically and aerobically *(data not shown).*

Binding isotherms were fit using Mathematica (version 8 Wolfram Research) to a model that involved the mass action equations for the three competing equilibria:

(1) $$P+D_{f}\underset{K_{1}}{↔}\;PD_{f}$$

(2) $$P+D\underset{K_{2}}{↔}PD$$

(3) $$PD_{f}+D\underset{K_{3}}{↔}\;PD+D_{f}$$

Where P is the protein (*Hp*ArsR-P), D_f_ is fluorescently labeled DNA, and D represents unlabeled DNA. The value for K_1_ was determined from the forward titrations and thus used as a known parameter for the fit. Mathematica software was used to combine Equations (1)–(3) and to solve the resulting cubic equation in terms of *PD_f_* using non-linear, least squares analysis. All titrations were carried out in triplicate (Dosanjh et al., 2009).

### Statistical analysis

Statistical analysis on qRT-PCR and flow cytometry data was conducted using GraphPad Prism 6.01 (GraphPad Software, La Jolla, CA, USA). A One-Way ANOVA was used to compare basal levels of expression in Δ*nikR*, Δ*arsS*, and ΔarsS/*nikR* to WT. A Two-Way ANOVA with Tukey's correction for multiple comparisons was used to analyze fold differences in expression between WT *H. pylori*, Δ*nikR*, Δ*arsS*, and ΔarsS/*nikR*. Similarly, mean fluorescent intensity (MFI) values obtained from flow cytometry were assessed using a two-way ANOVA with Tukey's correction for multiple comparisons. A *p* < 0.05 was considered significant.

## Results

### Regulation of *P_ureA_* by *Hp*NikR

*Hp*NikR positively regulates the expression of urease by binding to a partially palindromic sequence located on the *ureA* promoter (*P_ureA_*) (Dosanjh et al., 2007, 2009; Evans and Michel, 2012). The DNA sequence that *Hp*NikR recognizes and binds to is AT rich and consists of seven base pairs separated by an eleven base pair linker sequence that is not thought to be directly involved in the protein/DNA recognition event (Figures 1B,C). When either half of the palindrome is mutated to all cytosines, the *in vitro* affinity of *Hp*NikR for the DNA sequence is diminished by 3 orders of magnitude from a K_d_ of 8.0 ± 1 nM to a K_d_ of 1.0 ± 0.09 or 4.9 ± 0.8 μM for the WT/C or C/WT mutants, respectively (Dosanjh et al., 2009; West et al., 2012). When both halves of the palindrome are mutated to all cytosines, DNA binding is fully abrogated (Dosanjh et al., 2009). Thus, both sides of the recognition palindrome appear to be important for *Hp*NikR/DNA binding.

To determine if these *in vitro* identified DNA sequence requirements are also observed *in vivo*, a series of Green Fluorescent Protein (GFP) reporter constructs in which the DNA recognition sequence within the *ureA* promoter was varied, to mirror the modifications that were studied *in vitro*, were created. These constructs, named WT/WT, WT/C, C/WT, and C/C, were unmodified or modified at each palindrome as indicated in Table 2. To measure the effect of these modifications on the nickel-dependent transcription of urease controlled by endogenous *Hp*NikR, expression of GFP was monitored in *H. pylori* strain G27 and an isogenic Δ*nikR* strain grown in the presence of increasing Ni(II) concentrations.

**Figure 2:**
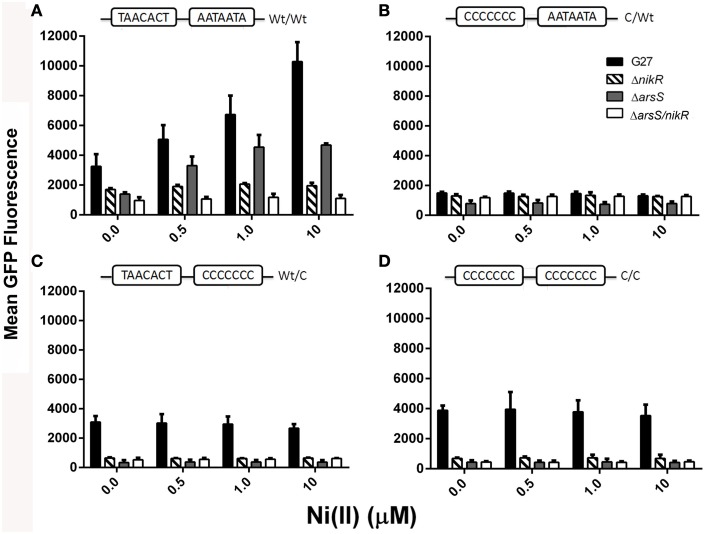
**Regulation of** ***P_ureA_*****by** ***Hp*****NikR and** ***Hp*****ArsRS**. The mean GFP expression for the *P_ureA_* WT and mutant constructs expressed in the various parental strains is shown. Each panel corresponds to a different P_ureA_ mutant construct that is indicated at the top of each panel: **(A)**- WT/WT, **(B)**- C/WT, **(C)**- WT/C, and **(D)**- C/C. Shading is as indicated: WT G27 (black bars), Δ*nikR* (cross bars), Δ*arsS* (gray bars), and Δ*arsS/nikR* (white bars) in NiSO_4_ supplemented (0, 0.5, 1, 10 μM) growth media. Flow cytometry was performed 3–5 times for each strain-reporter plasmid combination. Bars represent the mean GFP fluorescence and the error bars indicate the standard deviation.

Visual inspection of the strains by fluorescence microscopy showed that fluorescence was only observed in strains carrying constructs in which the *ureA* promoter was fused to GFP; promoterless controls showed no fluorescence (data not shown). Quantitative analysis of GFP expression was achieved using flow cytometry, as described in the materials and methods. Maximum GFP expression was observed for the WT/WT reporter construct carried in the wildtype *H. pylori* strain. This was the only reporter construct for which Ni(II) dependence was observed; a statistically significant concentration-dependent increase in GFP expression was observed as the Ni(II) concentration was increased (Figure 2 and Tables 3, 4). In the wildtype *H. pylori* strain background, GFP expression levels for the WT/C and C/C reporter constructs were similar to the GFP expression levels observed for the WT/WT reporter construct when the media was not supplemented with Ni(II) (Figure 2 and Table 4). In contrast, basal levels of *ureA* expression in the C/WT background were significantly lower than WT/WT in the wildtype *H. pylori* strain (*p* < 0.0001). At high Ni (II) concentrations, the reporter constructs containing mutation of the palindrome half sites (WT/C and C/WT) as well as full site (C/C) exhibited decreased GFP expression levels when compared to the unmodified WT/WT construct (Table 4). *En masse*, the fact that we observed high basal levels of expression of *ureA* with some of the mutant constructs was unexpected; our *in vitro* findings suggest that mutation of both sides of the palindrome completely abrogate *Hp*NikR binding at the *ureA* promoter (Dosanjh et al., 2009). Given that nickel responsiveness was completely lost for each mutated promoter construct (Figure 2 and Table 4) and because increasing nickel concentrations are known to result in increased *Hp*NikR activity, this finding suggested that another regulator may play a role in *ureA* expression under the *in vivo* conditions examined in these experiments.

**Table 3:** **Mean GFP fluorescence in normal and 10 μM NiSO_4_ supplemented media**.

**Strain**	**WT/WT**			**WT/C**			**C/WT**			**C/C**		
	N. media^*^	**+ Ni(II)^†^**	**Ni(II) dependent**	N. media^*^	**+ Ni(II)^†^**	**Ni(II) dependent**	N. media^*^	**+ Ni(II)^†^**	**Ni(II) dependent**	N. media^*^	**+ Ni(II)^†^**	**Ni(II) dependent**
G27	3252	10282	yes	3094	2671	no	1498	1309	no	3890	3538	no
Δ*nikR*	1708	1959	no	642	643	no	1298	1259	no	686	686	no
Δ*arsS*	1399	4688	yes	332	368	no	782	785	no	439	410	no
Δ*arsS/nikR*	963	1110	no	527	618	no	1186	1266	no	446	465	no

* All values are represented as mean GFP fluorescence.

† 10 μM NiSO_*4*_ added to media.

**Table 4:** **Statistical analysis of mean GFP fluorescence**.

**WT/WT *ureA* promoter construct with increasing nickel concentrations**					
	**Summary**	**Adjusted *p*-value^#^**		**Summary**	**Adjusted *p*-value^#^**
G27 WT			Δ*arsS*		
0 uM Ni vs. 0.5 uM Ni	^**^	0.0023	0 uM Ni vs. 0.5 uM Ni	^*^	0.0171
0 uM Ni vs. 1.0 uM Ni	^****^	<0.0001	0 uM Ni vs. 1.0 uM Ni	^****^	<0.0001
0 uM Ni vs. 10 uM Ni	^****^	<0.0001	0 uM Ni vs. 10 uM Ni	^****^	<0.0001
Δ*nikR*			Δ*arsS/nikR*		
0 uM Ni vs. 0.5 uM Ni	ns	0.9882	0 uM Ni vs. 0.5 uM Ni	ns	0.9984
0 uM Ni vs. 1.0 uM Ni	ns	0.9362	0 uM Ni vs. 1.0 uM Ni	ns	0.9857
0 uM Ni vs. 10 uM Ni	ns	0.9761	0 uM Ni vs. 10 uM Ni	ns	0.995
WT G27 basal level GFP expression with varying *ureA* promoter constructs			WT G27 GFP expression following 10 μM Ni^2+^ exposure with varying *ureA* promoter constructs		
WT/WT vs. C/WT	^*^	0.0271	WT/WT vs. C/WT	^****^	<0.0001
WT/WT vs. WT/C	ns	0.9935	WT/WT vs. WT/C	^****^	<0.0001
WT/WT vs. C/C	ns	0.711	WT/WT vs. C/C	^****^	<0.0001
Basal level GFP expression from WT *ureA* promoter in varying strain backgrounds			GFP expression following 10 μM Ni^2+^ exposure from WT *ureA* promoter in varying strain backgrounds		
G27 WT vs. Δ*nikR*	^****^	<0.0001	G27 WT vs. Δ*nikR*	^****^	<0.0001
G27 WT vs. Δ*arsS*	^****^	<0.0001	G27 WT vs. Δ*arsS*	^****^	<0.0001
G27 WT vs. Δ*arsS/nikR*	^****^	<0.0001	G27 WT vs. Δ*arsS/nikR*	^****^	<0.0001
Basal GFP expression			GFP expression following 10 μM Ni^2+^ exposure		
C/WT			C/WT		
G27 WT vs. Δ*nikR*	ns	0.8802	G27 WT vs. Δ*nikR*	ns	0.9994
G27 WT vs. Δ*arsS*	ns	0.0567	G27 WT vs. Δ*arsS*	ns	0.5864
G27 WT vs. Δ*arsS/nikR*	ns	0.6586	G27 WT vs. Δ*arsS/nikR*	ns	0.9996
WT/C			WT/C		
G27 WT vs. Δ*nikR*	^****^	<0.0001	G27 WT vs. Δ*nikR*	^***^	0.0001
G27 WT vs. Δ*arsS*	^****^	<0.0001	G27 WT vs. Δ*arsS*	^****^	<0.0001
G27 WT vs. Δ*arsS/nikR*	^****^	<0.0001	G27 WT vs. Δ*arsS/nikR*	^***^	0.0001
C/C			C/C		
G27 WT vs. Δ*nikR*	^****^	<0.0001	G27 WT vs. Δ*nikR*	^****^	<0.0001
G27 WT vs. Δ*arsS*	^****^	<0.0001	G27 WT vs. Δ*arsS*	^****^	<0.0001
G27 WT vs. Δ*arsS/nikR*	^****^	<0.0001	G27 WT vs. Δ*arsS/nikR*	^****^	<0.0001

# Adjusted p-value, p-value corrected for multiple comparisons using Tukey's multiple comparisons test. ns, non-significant.

* p-value ≤ 0.05;

** p-value ≤ 0.01;

*** p-value ≤ 0.001;

**** p-value < 0.0001

To specifically examine the contribution of *Hp*NikR to the observed levels of *ureA* expression, the same four promoter constructs were next examined in an isogenic Δ*nikR* strain. Interestingly, GFP expression was not entirely abrogated for the WT/WT promoter though expression was not nickel responsive (Figure 2 and Table 4). Once again, this suggests that the GFP expression observed in the wildtype strain is regulated by *Hp*NikR as well as another regulatory factor (Figure 2). Mutating either side of the *Hp*NikR binding sequence in the *P_ureA_* promoter abrogated the Ni (II) response and resulted in varying effects on basal levels of *ureA* expression. Mutation of the right half of the recognition sequence (WT/C) resulted in a significant decrease in expression (*p* = 0.0021) as compared to the WT/C construct in the wildtype strain. Likewise, disruption of both segments of the palindrome (C/C) resulted in a dramatic decrease in *ureA* expression in the Δ*nikR* mutant (Figure 2) that was similar to the decrease observed in the WT/C construct (Table 4). Interestingly, mutating the left half (C/WT) of the recognition sequence did not lead to a significant reduction in *ureA* expression (*p* = 0.4408) as compared to the C/WT construct in the wildtype strain. When considered together, these findings suggest that an additional regulatory factor is involved in urease transcription. Moreover, given that *in vitro* studies have clearly shown that *Hp*NikR does not bind to the C/C mutant construct (Dosanjh et al., 2009), the demonstrated decrease in transcription when *Hp*NikR is absent in the strain carrying the C/C mutant construct suggests that *Hp*NikR has a hitherto unidentified indirect role in *P_ureA_* transcription.

### Regulation of *P_ureA_* by *Hp*ArsR

The observation that *P_ureA_* transcription occurs for the WT/C and C/WT GFP reporter constructs in the presence and absence of *Hp*NikR independent of nickel availability suggested that another factor regulates *ureA* transcription in these conditions. The two-component system *Hp*ArsRS has been shown to mediate pH-responsive urease expression (Pflock et al., 2005, 2006a,b; Wen et al., 2006, 2007). Furthermore, the *Hp*ArsR binding site within the *ureA* promoter partially overlaps the site recognized by *Hp*NikR (Figures 1B,C). To determine if *Hp*ArsR was responsible for the observed urease expression, Δ*arsS* and Δ*arsS/nikR* deletion strains of *H. pylori* were constructed and GFP fluorescence was measured for each of the *P_ureA_* constructs. Of note, this strategy was chosen since *Hp*ArsR was previously shown to be essential and cannot be deleted (Beier and Frank, 2000; McDaniel et al., 2001); however, *Hp*ArsR regulates urease in its phosphorylated form (*Hp*ArsR-P) (Pflock et al., 2005). Therefore, deletion of *Hp*ArsS effectively inactivates *Hp*ArsR dependent regulation of urease since *Hp*ArsS is required for *Hp*ArsR phosphorylation (Pflock et al., 2005, 2006a).

To dissect the role of *Hp*ArsR (Δ*arsS*) in *ureA* expression, transcription was measured for each of the *P_ureA_* constructs. As expected, because *Hp*NikR is present in the Δ*arsS* strain background, the WT/WT promoter showed Ni(II) dependent GFP expression. However, the relative amounts of GFP expression were significantly lower in this strain background as compared to the wildtype strain (Table 4): for example, a mean GFP fluorescence of 4688 fluorescence units was measured for the Δ*arsS* strain in 10 μM nickel as compared to 10,282 fluorescence units for wildtype under the same nickel concentrations (Figure 2 and Table 3). This finding suggests that under non-acidic conditions, *Hp*ArsR-P interacts cooperatively with *Hp*NikR to increase expression from the WT urease promoter. In the Δ*arsS/nikR* background, though a basal level of GFP expression is observed, this expression is nickel independent (Figure 2).

For the WT/C and C/C mutant promoters, the absence of endogenous *Hp*ArsS (and thus, *HpA*rsR-P) resulted in lower levels of GFP expression as compared to those observed in the wildtype strains (*p* < 0.0001 for both). In each case, GFP expression was unaffected by nickel concentration. Similar to what was seen in the Δ*nikR* background, while the WT/C and C/C promoters resulted in decreased expression, the decrease in *ureA* expression in the C/WT background was not significant as compared to the same construct carried in the wildtype strain (Table 4). Furthermore, basal levels of *ureA* expression in Δ*nikR* and Δ*arsS* strains were similar regardless of which *ureA* promoter was present (Table 4). In the Δ*arsS/nikR* background, the WT/C and C/WT mutant promoters showed levels of expression similar to those observed in the Δ*nikR* background. As was observed in the Δ*nikR* single mutant background, none of these promoter constructs was nickel responsive in the double mutant background (Figure 2 and Table 3). In the Δ*arsS* and Δ*arsS/nikR* backgrounds, the C/C promoter fusion produced low levels of GFP expression that were slightly less than the levels observed in the Δ*nikR* background. Significant GFP expression was only observed for this construct in the wildtype strain. Taken *en masse*, these findings are consistent with cooperative interaction of *Hp*NikR and *Hp*ArsR-P at the *ureA* promoter to achieve maximal regulation of urease. Of note, the role of *Hp*ArsR-P in this regulation occurred in the absence of acidic pH, which is considered to be the major environmental signal controlling *Hp*ArsR activity (Pflock et al., 2005, 2006a,b; Wen et al., 2006, 2007).

**Figure 3:**
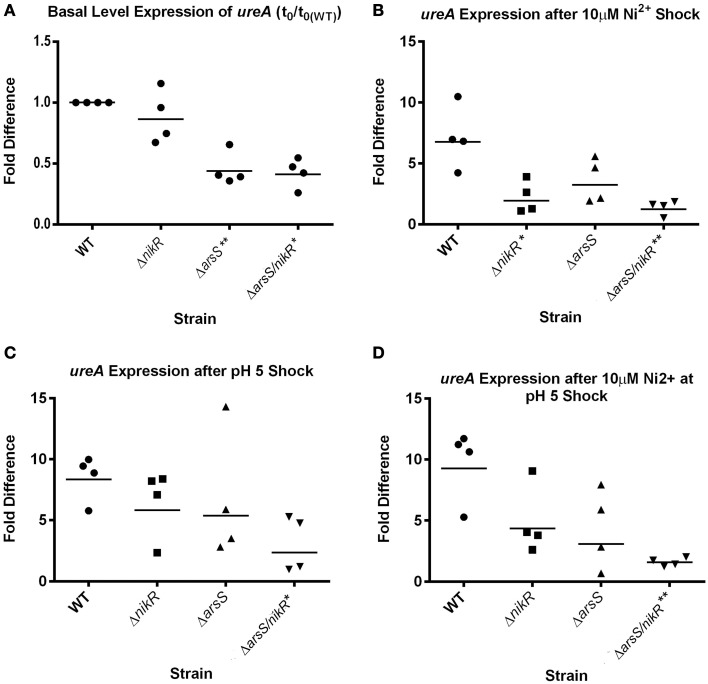
**Changes in** ***ureA*** **expression in response to nickel and low pH**. qPCR using *ureA* specific primers was performed on cDNA generated from WT, Δ*nikR*, Δ*arsS*, and Δ*arsS/nikR* strains exposed to 10 μM Ni^2+^, pH 5.0 or both stress conditions for 90 min following 18 h of growth in normal media. Relative gene expression was calculated using the 2^-ΔΔCT^ method. **(A)** The basal levels of *ureA* expression (relative to WT); **(B)** Changes in *ureA* expression following shock with 10 μM Ni^2+^; **(C)** Changes in *ureA* expression following shock with pH 5.0; **(D)** Changes in *ureA* expression following shock with 10 μM Ni^2+^ at pH 5.0. Four biologically independent replicates of these experiments were conducted, and each dot represents the fold difference from one replicate with bars representing the geometric mean fold difference. ^*^p < 0.05 compared to WT, ^**^p < 0.01 compared to WT.

To further investigate the cooperative regulation of *ureA* by *Hp*NikR and *Hp*ArsR-P and to confirm that the use of plasmid based transcriptional fusions was not artificially affecting our results, we next directly assessed *ureA* expression directly from the chromosome via qPCR analysis on RNA extracted from WT, Δ*nikR*, Δ*arsS*, and Δ*arsS/nikR H. pylori* strains. We assessed *ureA* expression in strains that were exposed to normal growth media as well as to medias that were 1) supplemented with excess nickel (10 μM), 2) adjusted to acidic pH (pH 5.0), or 3) supplemented with excess nickel and adjusted to acidic pH (10 μM + pH 5.0). Comparison of basal levels of *ureA* expression between the WT and mutant strains under normal growth conditions showed that there was little to no difference in *ureA* expression between WT and Δ*nikR* (*p* = 0.319); this was expected based on previous data (Ernst et al., 2005). Conversely, a statistically significant decrease in basal *ureA* expression was seen in the Δ*arsS* (*p* = 0.009) and Δ*arsS*/*nikR* (*p* = 0.0116) strains (Figure 3A). As with the data obtained with the GFP fusions (Figure 2), these data suggest that *Hp*ArsRS is necessary for maximal expression of *ureA* under normal growth conditions.

Following exposure to 10 μM Ni^2+^, *ureA* expression was strongly upregulated in WT *H. pylori* (approximately six-fold). However, the extent of *ureA* upregulation was significantly decreased in the Δ*nikR* strain (*p* = 0.0159). Although not significant, a decrease in Ni-dependent upregulation of *ureA* was also observed in the Δ*arsS* strain background (three-fold increase compared to six-fold in WT) (Figure 3B). Additionally in the Δ*arsS*/*nikR* double mutant there was no change in *ureA* expression upon exposure to nickel (Figure 3B). These data suggest that both *Hp*NikR and *Hp*ArsRS are required for maximal expression of *ureA* upon nickel stress. This point is further supported by the fact that the observed difference in nickel dependent *ureA* expression between Δ*nikR* and Δ*arsS* was not statistically significant (*p* = 0.5529). Similarly, upon exposure to acidic pH, *ureA* expression was increased approximately eight-fold in the WT strain, six-fold in Δ*nikR*, five-fold in Δ*arsS* but only two-fold in the Δ*arsS*/*nikR* double mutant (Figure 3C). A statistically significant difference in *ureA* expression under low pH was only observed when comparing WT and the Δ*arsS*/*nikR* double mutant (*p* = 0.0467). Given that similar levels of *ureA* expression were observed in both the Δ*nikR* and Δ*arsS* strains, this suggests that both regulatory proteins are necessary for maximal expression in the low pH environment; thus, *Hp*NikR appears to play a previously unknown role in the acid responsive regulation of *ureA* (Figure 3C). Lastly, changes in *ureA* expression were monitored following simultaneous exposure to excess nickel and low pH. Again, the largest increase in expression was observed in the WT strain background (nine-fold). Although not statistically significant, *ureA* expression was only moderately increased in the Δ*nikR* (four-fold) and Δ*arsS* (three-fold) strains (Figure 3D). Of note, under these conditions, Δ*nikR* and Δ*arsS* were not significantly different from each other (*p* = 0.8729) or from the Δ*arsS*/*nikR* double mutant (*p* = 0.1722 and *p* = 0.4847, respectively). However, given that no change in *ureA* expression was observed for the Δ*arsS/nikR* double mutant, *ureA* expression in the Δ*arsS*/*nikR* double mutant was significantly different from WT (*p* = 0.0059) (Figure 3D). *En masse*, these data support the notion that both *Hp*NikR and *Hp*ArsRS are important for regulation of *ureA* expression under normal conditions as well as in low pH and nickel supplemented environments.

### Fluorescence anisotropy to measure *Hp*ArsR-P/*P*_*ureA*_ binding *in vitro*

**Figure 4:**
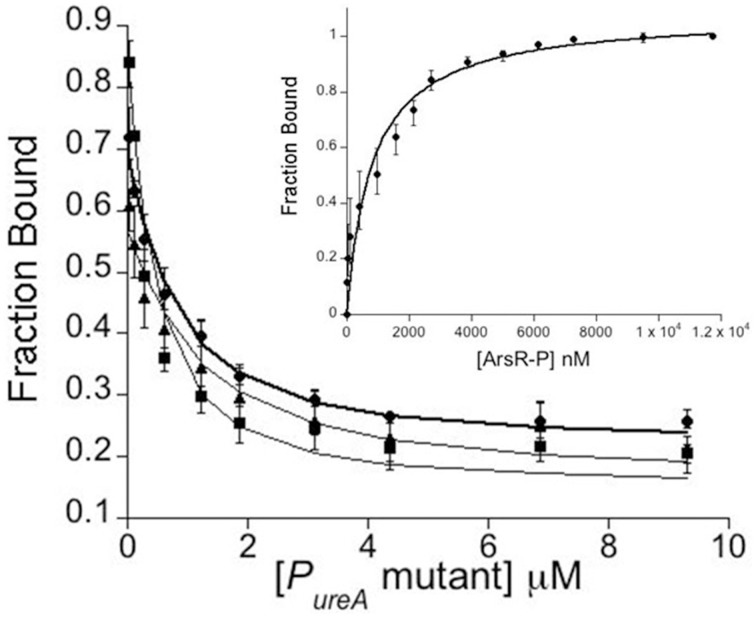
***Inset*** **Direct fluorescence anisotropy (FA) titration between** ***Hp*****ArsR-P and fluorescein labeled Wt/Wt** ***P**_**ureA**_**-F***. The data are fit to a 1:1 binding equilibrium. *Main Figure:* competitive titrations of *Hp*ArsR-P with P*_ureA_* mutants: • *P_ureA_*Wt/C, ■ *P_ureA_* C/Wt, and ▲ *P_ureA_* C/C into 5 nM P*_ureA_*-F and 1.5 μM *Hp*ArsR-P. The data are fit to a competitive binding equilibrium. The data shown are the average of three sets of binding data. All FA experiments were performed in 50 mM Tris-HCl, 5 mM MgCl_2_, 100 mM KCl, 5 mM TCEP, pH 7.5 and 25°C.

Based on previous DNase footprinting studies, *Hp*ArsR-P binds to two regions of the *P_ureA_* promoter (Pflock et al., 2005). These two operators are made up of 41 and 57 base pairs, respectively, and the larger operator sequence includes bases recognized by *Hp*NikR (Figures 1B,C). Based upon our *in vivo* results that suggest co-regulation of the *ureA* promoter by *Hp*ArsR and *Hp*NikR, the *in vitro* DNA binding properties of *Hp*ArsR-P for the *P_ureA_* sequences recognized by *Hp*NikR were measured using fluorescence anisotropy (FA), which is an approach that has been successfully used to measure *Hp*NikR/P*_ureA_* binding (Dosanjh et al., 2007, 2009; Evans and Michel, 2012; West et al., 2012). The FA data for *Hp*ArsR-P with *P_ureA_* WT/WT, WT/C, C/WT, and C/C showed comparable binding isotherms (Figure 4), with K_d_s of 17 ± 2.0, 24 ± 0.5, 20 ± 0.6, and 23 ± 0.7 nM respectively. These findings show that *Hp*ArsR-P can bind to the same *P_ureA_* promoter sequence as *Hp*NikR *in vitro*, and it can also bind to sequences in which the palindrome recognized by *Hp*NikR is altered. *Hp*ArsR did not exhibit any DNA binding (data not shown).

## Discussion

*Hp*NikR regulates urease production by binding to and activating transcription of *P_ureA_* (van Vliet et al., 2002, 2004a; Abraham et al., 2006; Dosanjh et al., 2007). The details of the protein/DNA binding interaction have been systematically studied *in vitro* and a key partially palindromic recognition sequence has been identified (van Vliet et al., 2002, 2004a; Abraham et al., 2006; Dosanjh et al., 2007). When either half-site of the recognition palindrome is modified to all cytosines, the affinity of *Hp*NikR for the DNA is significantly reduced, while mutation of both half sites to all cytosines abrogates binding (Dosanjh et al., 2009). Those findings led us to conclude that these half-sites were the recognition elements required for *Hp*NikR-mediated activation of *ureA* expression. Herein, we present studies designed to translate our previous *in vitro* findings to the *in vivo* conditions present within the *H. pylori* cell. These *in vivo* findings revealed that additional factors are involved in the regulation of urease. Specifically, maximal activation of *ureA* transcription required the *Hp*ArsRS two component system in addition to *Hp*NikR.

The initial suggestion that a second factor may regulate *ureA* transcription came from GFP reporter assays of the *P_ureA_* WT/C and C/WT half-site mutants. Some level of nickel-independent GFP expression was still observed for all *P_ureA_* constructs; though, the levels of expression were only nickel dependent in WT/WT (Figure 2 and Table 4). Interestingly, while P*_ureA_* transcription was significantly decreased for the C/WT half site mutation, WT/C and C/C promoter mutations yielded similar levels of GFP expression to WT/WT in the wildtype *H. pylori* strain background (Figure 2 and Table 4). These high levels of expression suggested that a second factor was involved in regulating *ureA* transcription *in vivo*. This hypothesis was further supported by the levels of *ureA* expression observed in the Δ*nikR* strain background. For the *P_ureA_* half-site mutant, C/WT, the level of *ureA* transcription was similar to that observed in the wildtype strain while for the WT/C mutant, the level of *ureA* transcription was approximately five-times less than in the wildtype strain. Additionally, expression of the C/C mutant construct was greatly diminished (approximately six times), indicating that the high level of expression seen for this construct in the wildtype strain background was *Hp*NikR dependent. However, *in vitro* protein/DNA binding data has clearly demonstrated that *Hp*NikR is unable to physically interact with this altered C/C DNA target. Thus, the role that *Hp*NikR plays in regulating the C/C target appears to be indirect.

Given these data, we sought to identify the other factor required for proper regulation of *ureA* transcription at neutral pH (pH 7.5). The *Hp*ArsRS two-component system is also known to control urease expression in *H. pylori*. However, this system, which is responsive to pH, is thought to function primarily under acidic conditions (Marcus et al., 2012). The data presented in this study, demonstrate that *Hp*ArsRS functions with *Hp*NikR to properly regulate urease expression at neutral pH. Within the *Hp*ArsRS two-component system, *Hp*ArsS functions as the histidine kinase, and *Hp*ArsR functions as the cognate response regulator. Upon sensing acidic pH, *Hp*ArsS phosphorylates *Hp*ArsR, which results in activation of the regulator (Pflock et al., 2006a; Joseph and Beier, 2007; Gupta et al., 2009; Muller et al., 2009). Interestingly, *Hp*ArsS is not essential for *H. pylori* survival, but *Hp*ArsR is essential (Schar et al., 2005). This suggests that the non-phosphorylated form of *Hp*ArsR regulates some essential component within the *H. pylori* cell. *Hp*ArsR has been shown to bind some target promoters in its non-phosphorylated form, while it only binds other promoters in its phosphorylated form (Wen et al., 2006). The *ureA* promoter is known to be bound and regulated by the phosphorylated form of *Hp*ArsR (Pflock et al., 2005). Thus, deletion of *arsS* from G27 results in an inactive *Hp*ArsR response regulator in terms of regulation of the *ureA* promoter. Using this strain we found that although expression of the WT/WT fusion was still nickel-dependent, the expression levels were significantly lower at all Ni (II) concentrations tested than in the G27 background (Figure 2 and Table 4), suggesting a role for *Hp*ArsR-P in maximal expression from this promoter. Similarly, the WT/C half-site mutant, showed considerable reduction in P*_ureA_::gfp* expression in the absence *arsS*; once again indicating a role for *Hp*ArsR-P. Interestingly the C/WT half-site mutant appeared to lock the level of expression of *ureA* at a basal level regardless of the strain background examined. Conversely, the C/C mutation affected *ureA* transcription in all of the tested mutant strain backgrounds, suggesting concomitant regulation by both *Hp*NikR and *Hp*ArsRS. Additionally, qPCR analysis revealed that basal levels of *ureA* expression were significantly reduced in the absence of *arsS* (*p* = 0.009) but not *nikR* (*p* = 0.319) (Figure 3A). Further support for this idea comes from the analysis of *ureA* expression following exposure to nickel, low pH or both stressors combined. Regardless of the stressor, the highest levels of *ureA* expression were observed in the WT strain background, with each single deletion showing only moderate levels of *ureA* and the least amount of expression occurring in the double Δ*arsS*/*nikR* strain (Figure 3).

Taken together, these data support a model of *ureA* transcription that involves “cross-talk” between *Hp*NikR and *Hp*ArsRS to maximize induction of urease even under neutral conditions. Prior to this study, these two regulators were believed to function independently as *Hp*NikR is responsive to nickel levels and *Hp*ArsRS is responsive to acidic shock (Pflock et al., 2005). Therefore, the observation that *ureA* transcription is 2–2.5 times lower in the Δ*arsS* background is particularly compelling since, under these conditions, *Hp*NikR is present and functional. Surprisingly, the data presented here also indicate a role for *Hp*NikR in the response to acid stress; previous reports suggested that the acid-induced increase in *ureA* expression was independent of NikR (Pflock et al., 2005). Using qualitative primer extension, Pflock et al. showed that there were similar increases in *ureA* expression upon exposure to pH 5.0 in strains with and without *nikR* (Pflock et al., 2005). However, in this work, we observed that the increase in *ureA* expression was less than that of wildtype in the Δ*nikR* mutant strain. The differences in our data and the previously published work could be due to differences in assay sensitivities (primer extension vs. qPCR) as well as differences in exposure to stress conditions. In the previous work, the bacteria were exposed to low pH for 60 min as compared to our 90 min exposure. Perhaps, a longer observation period following exposure to low pH allows for better measurement of the transcriptional changes in response to the stressor. Our data fully support the model that both *Hp*NikR and *Hp*ArsRS are necessary for maximal levels of *ureA* expression in response to low pH regardless of nickel concentration.

Although, the discovery of cross-talk between *Hp*NikR and *Hp*ArsR is unexpected, crosstalk, involving *Hp*NikR, *Hp*ArsR, or other *H. pylori* regulatory proteins in general, is not unique. For example, *Hp*NikR and *Hp*Fur co-regulate *fur* transcription (Delany et al., 2001, 2005) and *Hp*ArsS and *Hp*FlgS work in concert to recruit and activate urease (Marcus et al., 2012). In addition, regulatory crosstalk among transcription factors has been observed within *E. coli*. Specifically, the transcription factors MarA and Rob of *E. coli*, which are involved in the response to chemical stressors consequently enabling antibiotic resistance, are co-regulated through transcriptional cross-talk with each other (Miller et al., 1994; Martin et al., 1996; Martin and Rosner, 1997; Michan et al., 2002; Schneiders and Levy, 2006; McMurry and Levy, 2010; Warner and Levy, 2010).

Though the *in vivo* transcriptional assays revealed a role of *Hp*ArsRS in regulation of *ureA* transcription, they did not demonstrate whether this role was a direct protein/DNA binding interaction, or an indirect effect via another, yet to be identified, factor. As previous studies have shown a requirement of phosphorylation for DNA binding at the *ureA* promoter (Pflock et al., 2005), a direct effect would likely involve *Hp*ArsR-P (Dietz et al., 2002). Using FA, we examined whether *Hp*ArsR-P directly bound to the four DNA targets (WT/WT, WT/C, C/WT, and C/C) by titrating *Hp*ArsR-P with fluorescently tagged DNA targets. A change in FA, indicative of binding, was observed for all four DNA targets when *Hp*ArsR-P was titrated. No DNA binding was observed when the control non-phosphorylated *Hp*ArsR was studied. These data indicate that *Hp*ArsRS directly regulates *ureA* by binding to a 48-mer promoter sequence.

Together, the *in vitro* and *in vivo* data obtained for *Hp*ArsR-P provide valuable insight into the role of *Hp*ArsR from a biophysical and a biological perspective. The *in vitro* results that we obtained for *Hp*ArsR-P binding to *ureA* (and related mutants) teach us that *Hp*ArsR-P binds to the *ureA* promoter in a very different way than *Hp*NikR. *Hp*NikR requires a specific sequence (the pseudo-palindrome) for high affinity DNA binding (Dosanjh et al., 2009; Evans and Michel, 2012). In contrast, *Hp*ArsR-P does not require this specific pseudo-palindromic sequence for binding (i.e., there is equivalent binding when the pseudo-palindromic sequence is modified).

Two factors are often important when proteins bind to DNA: sequence and shape (Rohs et al., 2010; Parker and Tullius, 2011). For *Hp*NikR evidence indicates that sequence - the pseudo-palindromic sequence found within the *ureA* promoter - is important for binding; when the sequence is modified, binding is abrogated (Dosanjh et al., 2009; Evans and Michel, 2012). In contrast, for *Hp*ArsR-P the data indicate that the pseudo-palindromic sequence is not important; when the sequence is modified, binding is not affected (*vide supra*). This may mean that *Hp*ArsR-P/*ureA* binding is driven by shape (overall conformation/structure of the DNA), rather than sequence, or that the sequence recognized by *Hp*ArsR-P contains additional oligonucleotides than the sequence recognized by *Hp*NikR.

The *in vivo* data, for which the entire promoter is present (rather than the short stretch of DNA utilized in the *in vitro* binding studies), revealed that when the *ureA* sequence was modified to the C/WT sequence and *Hp*ArsR-P driven expression was measured, the expression decreased. In contrast, *Hp*ArsR-P driven expression for all of the other *ureA* sequences was not dramatically affected. This finding indicates that there must be another factor (or factors), beyond the direct recognition of *Hp*ArsR-P with the 48-mer *ureA* target sequences *in vitro*, that is important for *Hp*ArsR-P regulation of *ureA in vivo.*

The combination of *in vitro* and *in vivo* data presented here allows us to learn both about (i) the very specific binding event between *Hp*ArsR-P and *ureA* (48 mer), which informs on the fundamental biophysical basis of binding, and (ii) the overall regulation by *Hp*ArsR-P at the cellular level, which informs on the biological mechanism. We initiated these studies to determine how the *in vitro Hp*NikR/*ureA* binding that we had previously measured translated in an *in vivo* setting. The data indicate that the *in vivo* regulation is more complex that the *in vitro* protein/DNA binding. Furthermore, we identified *Hp*ArsR-P as another key player in this regulation. By then looking at *Hp*ArsR-P both *in vivo* and *in vitro*, we can draw the same conclusion regarding *Hp*ArsR-P function: that its role in regulation *in vivo* is more complex than its *in vitro* protein/DNA binding.

Prior to the work presented here, *Hp*NikR and *Hp*ArsRS were thought to function as independent regulators of transcription, with *Hp*NikR involved in regulation of urease in response to intracellular nickel availability and *Hp*ArsRS involved in urease regulation in response to intracellular acid shock (Pflock et al., 2005). Strikingly, the studies presented here reveal that *both Hp*NikR and *Hp*ArsR-P are necessary for maximum Ni(II) dependent regulation of urease *in vivo* as well as the maximal response to acid shock. The two proteins are not independent regulators but, instead, work cooperatively to regulate *ureA* transcription. This is the first time that “cross-talk” between *Hp*NikR and *Hp*ArsRS has been demonstrated, and further studies will be required to tease out the interactions that promote this cooperative effect.

## Author contributions

Conceived and designed the experiments: AW, BC, DM, SM. Strain Construction: AW, BC, HG, JG. Performed the experiments: AW, HG, BC, DH, OP, SS. Contributed reagents/materials/analysis tools: MF, DH. Wrote the paper: AW, BC, DM, SM.

### Conflict of interest statement

The authors declare that the research was conducted in the absence of any commercial or financial relationships that could be construed as a potential conflict of interest.
